# Tibial Condylar Valgus Osteotomy Combined With Inverted V‐Shaped High Tibial Osteotomy

**DOI:** 10.1002/atn2.70361

**Published:** 2026-11-08

**Authors:** Hiroshi Nakayama, Kazunori Yasuda, Eiji Kondo, Akihiko Yonekura, Toshiya Tachibana, Shinichi Yoshiya, Tomoya Iseki

**Affiliations:** ^1^ Department of Orthopaedic Surgery Hyogo Medical University Nishinomiya Hyogo Japan; ^2^ Knee Research Center Yagi Orthopaedic Hospital Sapporo Japan; ^3^ Center for Sports Medicine Hokkaido University Hospital Sapporo Japan; ^4^ Clinical Center for Sports Medicine Nagasaki University Hospital Nagasaki Japan; ^5^ Department of Orthopaedic Surgery Nishinomiya Kaisei Hospital Nishinomiya Hyogo Japan

## Abstract

In constructing surgical strategies for correction of varus deformity in osteoarthritic knees, both bony and intra‐articular deformities should be taken into consideration. Bony deformity is corrected by high tibial osteotomy (HTO), whereas intra‐articular deformity associated with increased joint line convergence angle can be addressed by tibial condylar valgus osteotomy (TCVO). In the TCVO procedure, the medial tibial joint line is elevated by an L‐shaped osteotomy. A surgical procedure combining open‐wedge HTO and TCVO has been reported before. Although this combined procedure can address both bony and articular deformities, correction of severe bony deformity by open‐wedge HTO with large wedge opening may induce problems of unacceptable joint line obliquity and increase in leg length. In this situation, inverted V‐shaped HTO reported by Aoki and Kondo can be an alternative option. The inverted V‐shaped HTO procedure is designed to combine hemiclosed and hemiopen osteotomies. However, the correction is made near the center of rotation of tibial angulation, and wedge width can be reduced compared with other HTO procedures; potential problems associated with conventional HTO can be avoided. In this paper, the surgical rationale and sequence of combined TCVO and inverted V‐shaped HTO for correction of severe varus deformity are described.

## Graphical abstract

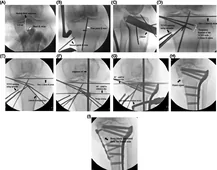

**VIDEO 1:** In this technical note, we present TCVO combined with iV‐HTO on the left knee. A 4‐cm oblique skin incision is made at the anteromedial part of the proximal tibia, and an L‐shaped incision is made to turn over the pes anserinus and the deep fascia. The superficial MCL is released at the distal insertion with an elevator. A short K‐wire was inserted at a point 2.5 cm distal to the medial intercondylar eminence. As a guide pin for osteotomy, the first 2.0‐mm guide K‐wire for the sagittal cut is inserted from the apex toward the lateral intercondylar eminence. Thereafter, a 2.0‐mm second guide K‐wire is inserted at 4 cm distal from the medial tibial joint line to the short K‐wire. The sagittal osteotomy through the ridge of the lateral intercondylar eminence is implemented using a chisel after K‐wire guidance under fluoroscopic control. In the lateral view, a chisel is first inserted from the anterior aspect of the tibia and extended superiorly and posteriorly, whereas the location of the chisel tip is carefully monitored under fluoroscopy. The second osteotomy, as the medial part of the L‐shaped osteotomy, is made using a chisel and a bone saw under direct vision. Before the deformity correction, two 1.5‐mm K‐wires are inserted from the lateral tibial condyle just below the joint line to the medial tibial condyle as transfixation. Then, the spreader is inserted into the transverse cut at the posterior cortex to avoid an increase in the posterior tibial slope. After the wedge opening, the medial bony fragment is temporarily fixed with two 2.0‐mm K‐wires from the medial side. For the subsequent iV‐HTO procedure, an anterolateral oblique skin incision is made beginning from the Gerdy tubercle and extending anterodistally along the anterior tibial crest by approximately 5 cm in length. A K‐wire is inserted from the lateral tibial edge 4 cm distal to the joint line, aiming toward the initially placed reference short K‐wire. A second K‐wire is inserted distally from the first K‐wire, with the distance corresponding to the preoperatively planned wedge width. The ascending cut behind the tibial tuberosity in a biplanar cut is made at approximately 110° to the horizontal plane, similar to the technique used in OWHTO, and the wedge is removed. After the completion of all osteotomies, valgus force is applied to achieve wedge closure. After the plate was temporarily fixed, the plate positioning and limb alignment were assessed. Fixation is achieved with a lateral locking plate using the MIPO technique. After the plate fixation, the medial spreader is removed, and the resected bone block from the lateral hemiwedge is grafted to the medial hemiopen‐wedge. The pes anserinus is sutured back by securing the cut edge to cover the osteotomy gap. The pes anserinus is sutured back by securing the cut edge to cover the osteotomy gap. Finally, fixation is completed using a small locking T‐plate over the pes anserinus. Fluoroscopic evaluation after the completion of all osteotomies confirms that TCVO effectively reduced the JLCA, resulting in the elimination of both medial and lateral instabilities. Video: Surgical sequence of TCVO combined with iV‐HTO. iV‐HTO, inverted V‐shaped high tibial osteotomy; JLCA, joint line convergence angle; MCL, medial collateral ligament; MIPO, minimally invasive plate osteosynthesis; OWHTO, open‐wedge HTO; TCVO, tibial condylar valgus osteotomy. Video content can be viewed at https://doi.org/10.1002/atn2.70361.

High tibial osteotomy (HTO) has been established as a joint preservation surgery for osteoarthritic knees through correction of coronal‐plane deformity.^1^, ^2^, ^3^ For correction of varus deformity in medial knee osteoarthritis, medial open‐wedge or lateral closed‐wedge osteotomy has been conventionally adopted as a surgical option. Considering the components of varus knee deformity, a primary factor is bony deformity occurring predominantly in the proximal tibia. In addition, increased joint line convergence angle (JLCA) associated with wear of articular cartilage and subchondral bone in the medial compartment can be an intra‐articular factor for varus knee alignment. Therefore, in constructing a surgical strategy for correction of severe varus deformity, considerations should be given to both bony and articular deformities. In such situations, correction of bony deformity by HTO alone is not enough to restore normal joint geometry and biomechanics.^4^, ^5^

Tibial condylar valgus osteotomy (TCVO) was first reported by Chiba et al. in 1990.^6^ This procedure is characterized by an L‐shaped osteotomy in the medial tibial condyle to correct intra‐articular varus deformity. With the intent of addressing both bony and articular deformities, combined TCVO and medial open‐wedge HTO (OWHTO) procedures were proposed for knees with severe varus deformity and a large JLCA, and their surgical procedure has been reported.^7^ Although this combined surgical approach can effectively correct the 2 components of varus deformity, large correction of bony deformity with medial OWHTO may lead to unacceptable joint line obliquity and leg lengthening. To cope with these problems, an inverted V‐shaped HTO (iV‐HTO) has been introduced to our practice as an alternative to conventional HTO. The surgical procedure and clinical outcomes of iV‐HTO were reported by Aoki et al.^8^ and Kondo et al.^6^, ^9^ The surgical concept of this procedure is to combine hemiclosed‐ and hemiopen‐wedge osteotomies with immediate local bone grafting. However, the correction is made near the center of rotation of tibial angulation, and the wedge width is smaller than in other HTO procedures; potential problems associated with conventional HTO can be avoided.

In this technical note, we describe our technique which combines modified TCVO and iV‐HTO to correct both bony and articular deformities in osteoarthritic knees with severe varus malalignment associated with large JLCA. The aim of this surgical option is to restore normal joint anatomy and biomechanics in this difficult‐to‐manage situation.

## SURGICAL TECHNIQUE

### Indication

This procedure is indicated for medial compartmental osteoarthritis of Kellgren‐Lawrence osteoarthritis grade ≥3 exhibiting excessive joint convergence with JLCA >4° in a weight‐bearing radiograph. This combined procedure can also be indicated for depression of the medial plateau as a sequel to Blount disease or other developmental disorders. In addition, the following criteria should be met: no lateral compartment involvement or systemic inflammatory arthritis, mechanical medial proximal tibial angle <87° and mechanical lateral distal femoral angle <90° in radiological measurements, and retained range of motion with extension loss <15° and flexion >130° in clinical examination.^9^

The choice between OWHTO and iV‐HTO depends on the amount of varus correction required. In cases in which the required opening width would exceed 15 mm with OWHTO or TCVO, iV‐HTO should be considered.

### Surgical Setting

The surgery is performed under general anesthesia. The patient is placed in the supine position on the operating table. A lateral thigh support is attached to the rail of the table to help with knee positioning and stress application during the procedure. The contralateral limb is positioned with the hip abducted to enable easier access to the medial aspect of the operative knee. A tourniquet is not used to monitor and control intraoperative bleeding.

### Arthroscopic Evaluation and Intra‐articular Procedure

Prior to osteotomy, arthroscopic examination is performed to evaluate the status of the intra‐articular structures, including the articular cartilage, meniscus, and synovia in all compartments. Subsequently, arthroscopic procedures such as debridement, synovectomy, meniscectomy, meniscal repair, and capsulodesis^10^ are performed as required. In addition, marginal osteophytes are arthroscopically harvested for subsequent use as bone chips to be grafted into the remaining defect at the osteotomy site.^9^, ^11^

### Osteotomy Procedure

As a first step of the surgical sequence, the fibular osteotomy was performed according to the method reported by Yasuda et al.^12^ A 4‐cm longitudinal incision is made on the skin at the lateral aspect of the leg, which is at the central portion between the fibular head and the lateral malleolus. The osteotomy line is created on a quasifrontal plane, inclined 25° to 30° relative to the long axis of the fibula. After HTO, 2 transosseous holes are created using a K‐wire, and a suture is passed through these holes. The suture is then securely tied 5 times after tibial osteotomy to maintain stable contact between the 2 osteotomized bone surfaces.

In the following step for tibial osteotomy, first, a 4‐cm oblique skin incision is made at the anteromedial part of the proximal tibia, and then, the periosteum of the osteotomy site is peeled off. An L‐shaped incision was made in the pes anserinus, and the deep fascia is turned over with sutures placed at the cut edge for later repair. The superficial medial collateral ligament is released at the distal insertion. The posterior soft tissue is stripped from the proximal tibia and retracted to protect the neurovascular bundle and avoid injuries to those structures during osteotomies. Tibial osteotomy is initiated with TCVO. To determine the location and level of the bony cut, a short K‐wire is inserted at a point 2.5 cm distal to the medial intercondylar eminence (Figure 1A). This point represents the hinge point of the TCVO and the apex of the iV‐HTO. As a guide pin for initial osteotomy in TCVO, a 2.0‐mm K‐wire for the sagittal cut is inserted from the apex toward the lateral intercondylar eminence. Then, the second 2.0‐mm K‐wire is inserted as a guide at 4 cm distal from the medial tibial joint line, directed to the apex point, as indicated by the short 2.0‐mm K‐wire, which represents the hinge point in TCVO (Figure 1B). The sagittal osteotomy through the ridge of the lateral intercondylar eminence is implemented using a chisel after K‐wire guidance under fluoroscopic control. In the lateral view, a chisel is first inserted from the anterior aspect of the tibia and directed superiorly and posteriorly, whereas the location of the chisel tip is carefully monitored during advancement under fluoroscopy (Figure 1C). The second osteotomy is the medial part of the L‐shaped osteotomy. A transverse cut is performed along the guide pin to complete the L‐shaped osteotomy in TCVO. Before the deformity correction, two 1.5‐mm K‐wires are inserted from the lateral tibial condyle just below the joint line toward the medial tibial condyle. These 2 K‐wires serve as transfixation to prevent separation at the osteotomy site during the deformity correction. The spreader is inserted into the transverse cut at the posterior wedge space to prevent an increase in the posterior tibial slope during the TCVO wedge opening. The wedge is opened until the coronal slope of the medial tibial plateau comes in line with that of the lateral tibial plateau (Figure 1D). During deformity correction in TCVO, the medial tibial condyle is elevated to come into contact with the lateral compartment. Adjustment of the JLCA to around 1° to 2° is preferable to achieve balanced intra‐articular alignment.^13^ The contact between the medial and lateral tibial plateaus precludes further opening. Thereafter, the whole‐leg alignment is assessed using an alignment rod on the fluoroscopic image. In general, TCVO alone is not capable of adequately correcting coronal alignment for the knees with severe varus deformity, and thus, concomitant HTO is required.^14^

**FIGURE 1:**
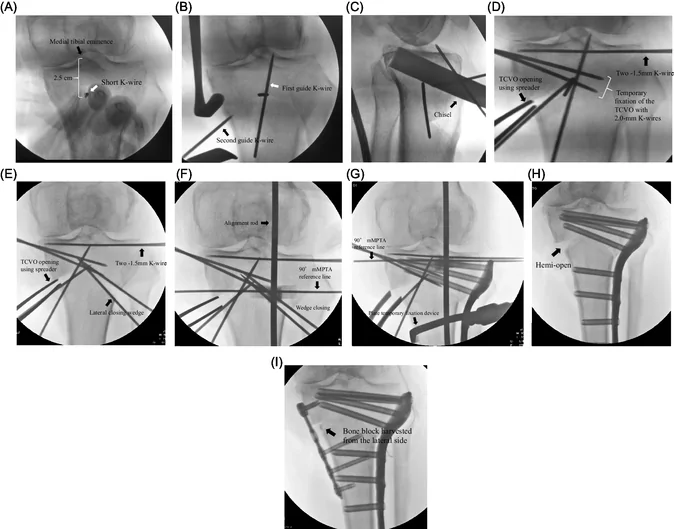
Surgical sequence of the combined TCVO and iV‐HTO procedure for the left knee. (A) The short K‐wire (white arrow) is inserted at a point 2.5 cm from the medial intercondylar eminence (black arrow). (B) The first K‐wire (white arrow) is inserted just lateral to the lateral intercondylar eminence. The second guide K‐wire is inserted 4 cm distal from the medial tibial joint line, directed to the hinge point indicated by the short K‐wire. (C) The chisel (black arrow) is advanced, whereas the location of its tip is carefully monitored on a lateral fluoroscopic image. The sagittal osteotomy is made using a bone saw and chisel following the first guide K‐wire. (D) Two 1.5‐mm K‐wires (black arrow) are inserted from the lateral tibial condyle just below the joint line to the medial tibial condyle as transfixation. After completing the L‐shaped osteotomy, a spreader is inserted into the transverse cut of the TCVO for wedge opening. After the wedge opening, two 2.0‐mm K‐wires are inserted from the medial side for temporary fixation of the TCVO. (E) Lateral closed‐wedge osteotomy is performed. (F) After lateral wedge closing, resultant leg alignment is assessed on a fluoroscopic image by confirming the location of the alignment rod at the tibial plateau level. (G) Plate fixation on the lateral side using a temporary fixation device. (H) Lateral plate fixation. (I) The resected bone block from the lateral side is grafted into the medial opening space (black arrow), and the medial support plate fixation is applied to the osteotomy site using a locking plate with a 3.5‐mm screw. (iV‐HTO, inverted V‐shaped high tibial osteotomy; mMPTA, mechanical medial proximal tibial angle; TCVO, tibial condylar valgus osteotomy.)

Then, iV‐HTO is performed according to the technique reported by Kondo et al.^11^ Before osteotomy, the medial bony fragment in TCVO is temporarily transfixed with 2 K‐wires from the medial side (Figure 1D). For the subsequent iV‐HTO procedure, an anterolateral oblique skin incision is made beginning from the Gerdy tubercle and extending anterodistally along the anterior tibial crest by approximately 5 cm in length. The fascia covering the tibialis anterior muscle is incised along the anterolateral edge of the tibial crest from a point just distal to the Gerdy tubercle and extends distally by approximately 5 cm. The tibialis anterior muscle attachment to the tibia is detached subperiosteally with an elevator. For the subsequent osteotomies in iV‐HTO, a radiolucent retractor (Osferion Biomaterials, Tokyo, Japan) is inserted between the tibia and the detached posterior structures to protect the neurovascular structures during the bony cuts. As a guide for the lateral hemiclosed‐wedge osteotomy, a K‐wire is inserted from the lateral tibial edge at 4 cm distal to the joint line under fluoroscopic control, aiming at the apex point as indicated by the initially placed reference short K‐wire. A second K‐wire is inserted distally from the first K‐wire, directed to the apex, with the distance corresponding to the preoperatively planned wedge width. After removal of the lateral closed wedge, a coronal ascending cut behind the tuberosity in a biplanar cut is made at approximately 110° to the horizontal plane, which is similar to the technique used in OWHTO. After the completion of all osteotomies, valgus force is applied to achieve correction of varus deformity by wedge closure (Figure 1G). When the optimal alignment is confirmed, plate fixation is performed. Fixation is achieved with a TriS lateral plate (Osferion Biomaterials) inserted through a subcutaneous tunnel along the lateral aspect of the tibia using the minimally invasive plate osteosynthesis technique. After the plate fixation, the medial spreader is removed, and the resected bone block from the lateral hemiwedge is grafted to the medial opening space. The pes anserinus is sutured back by securing the cut edge to cover the osteotomy gap. Finally, fixation is completed using a small T‐plate with 3.5‐mm screws (Osferion Biomaterials) placed over the pes anserinus, and K‐wires used for temporary fixation are removed. The wound is closed after placement of a surgical drain on the lateral side of the osteotomy. Pre‐ and postoperative radiographs and 3‐dimensional computed tomography are shown in Figures 2, 3 to 4.

**FIGURE 2:**
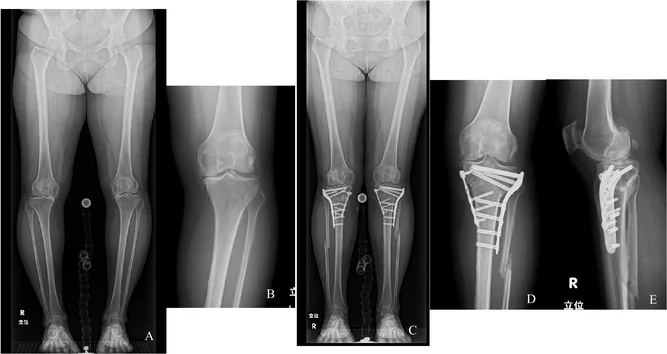
Pre‐ and postoperative plane radiographs (both knees) of a representative case. (A) Preoperative whole‐leg standing view. (B) Preoperative standing anteroposterior view of the left knee. (C) Postoperative whole‐leg standing view (6 months after surgery) of the left knee. (D) Postoperative anteroposterior view (6 months after surgery) of the left knee. (E) Postoperative lateral view (6 months after surgery) of the left knee.

**FIGURE 3:**
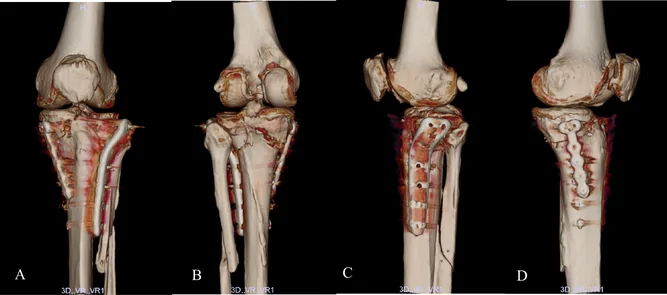
Postoperative 3D‐reconstructed CT (left knee) (1 week after surgery). (A) Anteroposterior view. (B) Posterior view. (C) Medial view. (D) Lateral view. (3D, 3‐dimensional; CT, computed tomography.)

**FIGURE 4:**
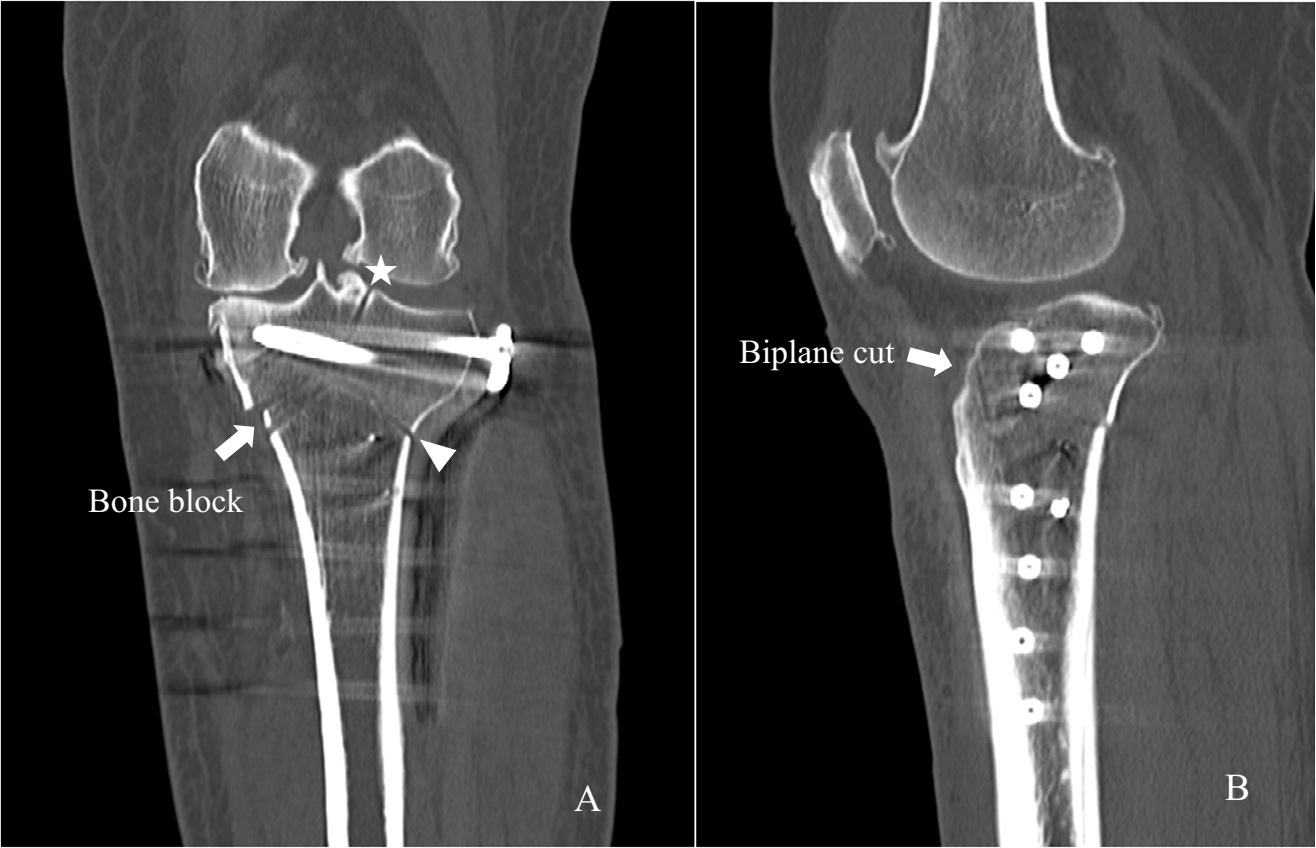
Postoperative CT images (left knee) (1 week after surgery). (A) Coronal view. The resected bone block from lateral side is grafted into the medial opening space (white arrow). White star: TCVO osteotomy line, arrowhead: iV‐HTO lateral hemiclosed‐wedge site. (B) Sagittal view. iV‐HTO is performed biplane cut (white arrow). (CT, computed tomography; iV‐HTO, inverted V‐shaped high tibial osteotomy; TCVO, tibial condylar valgus osteotomy.)

### Postoperative Rehabilitation

The operated knee is not immobilized after surgery, with range‐of‐motion exercise initiated as tolerated on the following day. Weight‐bearing is not allowed for the initial 2 weeks. Thereafter, partial weight‐bearing using crutches with a 20‐kg axial load is initiated with progression to full weight‐bearing at 4 weeks after surgery.

### Ethics Approval

The study was approved by the Review Board at Hyogo Medical University (No. 5297).

## DISCUSSION

Severe varus knee deformity is composed of bony deformity predominantly in the proximal tibia and the intra‐articular component associated with increased JLCA.^13^, ^15^ Therefore, correction of both bony and articular components should be considered in optimizing the surgical strategy for alignment correction. Isolated HTO in this situation can only address the bony component of the deformity, whereas instability and incongruity at an articular level remain to be present, leading to unpredictable or unfavorable surgical outcomes.^6^, ^7^, ^13^, ^15^

TCVO, which is an L‐shaped osteotomy in the medial tibial condyle, can restore the alignment at the articular level by levering up the medial tibial joint line and effectively correct the articular component of the varus deformity. Kuwashima et al. have proposed the TCVO procedure and reported that TCVO is capable of improving joint congruity and joint stability, though its efficacy in correction of varus limb alignment is limited.^7^ Therefore, in constructing a surgical approach to severely varus knees with both bony and articular deformities, combined procedures of TCVO and HTO can be a reasonable option.

Based on this consideration, we have proposed and reported combined TCVO and medial open‐wedge distal tuberosity tibial osteotomy procedures for correction of severe varus deformity.^16^ Medial open‐wedge distal tuberosity tibial osteotomy is a modification of medial OWHTO with the intent of avoiding the postoperative patella infra, which is an inherent shortcoming of the OWHTO^17^, ^18^ by conducting an osteotomy with the tibial tubercle attached to the bony fragment proximal to the wedge osteotomy.^16^ Although the proposed combined procedure may address both bony and articular components of the deformity, the open‐wedge osteotomy has a practical limit in the amount of correction because of the potential risks for unacceptable joint line obliquity and leg lengthening caused by large wedge opening.^9^, ^19^ Consequently, its indication is limited for severe varus knee deformity with a small preoperative mechanical medial proximal tibial angle.

The iV‐HTO procedure, which is classified as a neutral wedge osteotomy, has the potential to solve the disadvantages of OWHTO.^9^, ^11^ In correction of severe varus deformity located in the proximal tibia, conventional HTO procedures may bear a risk of undesirable change in the tibial length after surgery. In contrast, in the iV‐HTO procedure, correction is made at the center of rotation of tibial angulation, and the resected lateral hemiwedge bone is used for grafting to the medial gap. Consequently, the original tibial length and morphology can be preserved.^9^, ^11^ Therefore, this technique can be a reasonable alternative to other HTO procedures for knees with severe varus bony deformity.

There are some additional risks and pitfalls associated with the application of the combined procedure reported in this paper. First, postoperative local hemorrhage and limb swelling can be more pronounced than isolated HTO because of the increased bony cuts (additional osteotomies in TCVO). To avoid postoperative complications such as hematoma and compartment syndrome, a surgical drain is routinely used in our practice. Second, excessive correction of JLCA to the level of 0° in TCVO may result in joint stiffness and consequently impair the patient's satisfaction. Therefore, we consider that correction leaving a postoperative JLCA of approximately 1° to 2° is preferable. Moreover, double‐plate fixation is mandatory to ensure sufficient stability at the osteotomy sites and promote reliable bone healing, which may add to the surgical invasion. The pearls and pitfalls of this technique are shown in Table 1, and the advantages and disadvantages are presented in Table 2. The surgical procedure is shown in Video 1. TCVO combined with iV‐HTO can achieve optimal alignment correction for varus knee deformity with a large JLCA by addressing both bony and articular components of deformity. This surgical option can effectively improve the biomechanical environment of the knee while avoiding the potential risks of complications after HTO requiring a large wedge width.

**TABLE 1:** Pearls and Pitfalls of This Technique

**Pearls**	**Pitfalls**
Make an oblique skin incision to approach to the surgical area	During the sagittal osteotomy in TCVO, the location of the chisel tip must be carefully monitored on the lateral fluoroscopic image to avoid inadvertent neurovascular injuries
Insert a short hinge pin as a reference 2.5 cm distal from the medial intercondylar eminence, which represents the apex of TCVO and hinge point of iV‐HTO	Posterolaterally directed bony cut during the descending osteotomy may result in undesirable obliquity of the osteotomy plane
Make sagittal and horizontal bony cuts in the medial tibial condyle to complete TCVO following the guide K‐wires under fluoroscopic control	Excessive wedge opening at the transverse cut in TCVO may cause fracture of the hinge, leading to interfragment separation
Increased JLCA can be reduced by levering up the medial tibial plateau in TCVO. Optimal coronal alignment is achieved by adding iV‐HTO as a combined procedure	Correction with postoperative JLCA of approximately 1° to 2° is preferable, as overcorrection to 0° may result in joint stiffness and consequently impair the patient's satisfaction
The bone wedge harvested from the lateral hemiwedge in iV‐HTO is grafted into the medial hemiopening‐wedge site	Signs of postoperative compartment syndrome should be carefully monitored, especially in patients with large muscle volume
Double‐plate fixation can provide improved stability	

iV‐HTO, inverted V‐shaped high tibial osteotomy; JLCA, joint line convergence angle; TCVO, tibial condylar valgus osteotomy.

**TABLE 2:** Advantages and Disadvantages

**Advantages**	**Disadvantages**
Capability to correct both bony and articular deformities	Technically demanding procedure
Achievement of joint congruity and stability by TCVO	Prolonged surgical time
Prevention of patella infra by iV‐HTO	Increased number of bony cuts (2 osteotomies in TCVO and 3 osteotomies in iV‐HTO)
Avoidance of change in tibial length and morphology because of correction at the CORA in iV‐HTO	Repeated use of the alignment rod intraoperatively to assess coronal alignment
Wide application of iV‐HTO to the knees with advanced osteoarthritis	Requirements of fibular osteotomy and double plating, resulting in increased surgical invasiveness
No postoperative MCL tightness even after correction of severe varus deformity as a result of MCL release during iV‐HTO	Potential for increased postoperative hemorrhage and swelling requiring routine use of a surgical drain
Sustainable nature of iV‐HTO as an osteotomy technique because of local bone graft from lateral hemiclosing‐wedge to medial hemiopening gap	Full weight‐bearing not allowed until 4 weeks after surgery

CORA, center of rotation of tibial angulation; iV‐HTO, inverted V‐shaped high tibial osteotomy; MCL, medial collateral ligament; TCVO, tibial condylar valgus osteotomy.

## DISCLOSURES

The author (H.N.) declares the following financial interests/personal relationships, which may be considered potential competing interests: H.N. reports consulting fees from Osferion Biomaterials. The other authors (K.Y., E.K., A.Y., T.T., S.Y., T.I.) declare that they have no known competing financial interests or personal relationships that could have appeared to influence the work reported in this paper.
